# *Escherichia coli* Nissle 1917 enhances bioavailability of serotonin in gut tissues through modulation of synthesis and clearance

**DOI:** 10.1038/srep17324

**Published:** 2015-11-30

**Authors:** Jonathan Nzakizwanayo, Cinzia Dedi, Guy Standen, Wendy M. Macfarlane, Bhavik A. Patel, Brian V. Jones

**Affiliations:** 1 School of Pharmacy and Biomolecular Sciences, University of Brighton, Brighton, BN2 4GJ, United Kingdom; 2 Queen Victoria Hospital NHS Foundation Trust, East Grinstead, RH19 3DZ, United Kingdom

## Abstract

Accumulating evidence shows indigenous gut microbes can interact with the human host through modulation of serotonin (5-HT) signaling. Here we investigate the impact of the probiotic *Escherichia coli* Nissle 1917 (EcN) on 5-HT signalling in gut tissues. *Ex-vivo* mouse ileal tissue sections were treated with either EcN or the human gut commensal MG1655, and effects on levels of 5-HT, precursors, and metabolites, were evaluated using amperometry and high performance liquid chromatography with electrochemical detection (HPLC-EC). Exposure of tissue to EcN cells, but not MG1655 cells, was found to increase levels of extra-cellular 5-HT. These effects were not observed when tissues were treated with cell-free supernatant from bacterial cultures. In contrast, when supernatant recovered from untreated ileal tissue was pre-incubated with EcN, the derivative cell-free supernatant was able to elevate 5-HT overflow when used to treat fresh ileal tissue. Measurement of 5-HT precursors and metabolites indicated EcN also increases intracellular 5-HTP and reduces 5-HIAA. The former pointed to modulation of tryptophan hydroxylase-1 to enhance 5-HT synthesis, while the latter indicates an impact on clearance into enterocytes through SERT. Taken together, these findings show EcN is able to enhance 5-HT bioavailability in ileal tissues through interaction with compounds secreted from host tissues.

The human gastro-intestinal (GI) tract harbours a dense and complex microbial community, which undertakes a range of functions beneficial to the human host^1^,^2^,^3^,^4^,^5^. The pivotal role this gut microbiome is now believed to play in human health has led to a growing interest in strategies to manipulate its structure and function, to treat or prevent a spectrum of GI disorders. In this context much effort has been focused on probiotic microorganisms, defined as “live microorganisms which when administered in adequate amounts confer a health benefit on the host”^6^. Considerable evidence is now available to support the clinical efficacy of probiotics in treatment or prophylaxis of a range of maladies, not only those localised to the GI tract, but potentially also extra-intestinal disorders^7^,^8^,^9^,^10^,^11^,^12^,^13^.

Of the currently available probiotic bacteria, *E. coli* Nissle 1917 (EcN) is notable as the only Gram-negative species presently in use^11^. This gastroprotecitve *E. coli* strain (serotype O6:K5:H1) was first isolated from the faeces of a World War I soldier who, in contrast to others in his trench, was not affected by an outbreak of bacterial dysentery^14^. EcN now forms the active component of Mutaflor^®^ (Ardeypharm GmbH, Herdecke, Germany), a microbial probiotic drug that is marketed and used in several countries. Clinical trials have shown EcN to be effective for maintaining remission of ulcerative colitis (UC)^15^,^16^,^17^,^18^, for stimulation of the of the immune system in premature infants^19^, for treatment of infectious GI diseases^20^,^21^, for the relief of constipation^22^,^11^, and also treatment of Irritable Bowel Syndrome in some patients^23^. However, as with other probiotics, the mechanisms through which EcN exerts these beneficial effects, and interact with the human host, remain largely undefined.

An area of particular interest in this regard is the potential for probiotics to modulate synthesis, release, and reuptake of transmitters by epithelial endocrine cells (enterochromaffin cells, EC cells) in the gut. In particular, serotonin (5-Hydroxytryptamine or 5-HT) is an abundant gut associated transmitter, with EC cells of the gut epithelium generating ~90% of the total 5-HT pool extant in the human body^24^,^25^. The synthesis of 5-HT in EC cells begins with the conversion of tryptophan to the intermediate 5-hydroxytryptophan (5-HTP), catalysed by tryptophan hydroxylase-1 (Tph-1). Subsequently, 5-HTP is converted to 5-HT through the activity of aromatic L-amino acid decarboxylase (L-AADC), and the newly synthesised 5-HT is stored in vesicles before release following mechanical and/or chemical stimulation of the mucosa^25^,^26^. The majority of 5-HT released from EC is subsequently cleared into the neighboring enterocytes by the serotonin transporter (SERT), and rapidly metabolised to various end products, of which 5-hydroxyindole acetic acid (5-HIAA) is the most abundant^25^,^26^.

In the GI tract 5-HT interacts with a variety of serotonergic receptors located on intrinsic primary afferent neurons (IPANs), and regulates a variety of gut functions, including gut motility, ion secretion, and sensory functions^27^,^28^,^29^. Through sequestration by platelets, 5-HT synthesised in the GI tract is also distributed systemically, and contributes to regulation of global homeostatic processes, as well as key aspects of human development. Among these are roles of regulation of immune responses, bone development, and cardiac function^30^,^31^,^32^,^33^, as well as an influence on aspects of mood and behaviour^13^,^34^. In keeping with a crucial role in normal gut function, alterations in 5-HT signalling have been implicated in various diseases and disorders such as colon carcinoma, inflammatory bowel diseases (IBD), enteric infections, and functional disorders such as irritable bowel syndrome (IBS)^35^,^36^,^37^,^38^,^39^. Our own work has also recently linked alteration of 5-HT signalling with age related decline in gut function, including motility and the onset of chronic constipation^40^,^41^.

In terms of factors that may modulate 5-HT signalling, mounting evidence that the gut microbiome can modulate this pathway is of particular relevance to the development of probiotic-based interventions for GI disorders^13^,^42^,^43^. Recent studies using animal models have demonstrated a clear impact of gut-associated microbes on 5-HT signalling, both in the GI tract and systemically. In particular, germ free animals devoid of a gut microbiome exhibit significantly altered levels of 5-HT in the brain and peripheral circulation, and altered turnover of 5-HT^13^,^42^,^43^,^44^. Furthermore, members of the gut microbiota related to *Clostridia* (a dominant family of bacteria in the gut microbiome) have been shown to modulate 5-HT signalling through production of soluble metabolites that influence 5-HT synthesis, and through this mechanism, affect both GI motility as well as platelet aggregation^43^. Recent evidence also points to a role for 5-HT signalling in communication between the GI tract, the central nervous system and brain, and the gut microbiome (the microbiome-gut-brain axis), with manipulation of the gut microbiome associated with changes in aspects of host behaviour and mood through alteration of this dialogue^13^.

Taken together, the role of the serotonergic system in both local and systemic disease, the GI tract as the major site of 5-HT production in humans, and emerging evidence of gut microbiome mediated regulation of 5-HT bioavailability, point to manipulation of serotonergic signalling in particular as a prime target for development of probiotic interventions. However, relatively little is known regarding the impact of EcN or other species on 5-HT bioavailability or signalling in the GI tract. Therefore, the aim of this study was to evaluate the capacity for EcN treatment to influence regulation of the serotonergic system in intestinal tissues, using an *ex-vivo* co-culture model. In doing so we demonstrate the ability of EcN, but not a commensal *E. coli* strain (MG1655), to influence 5-HT signalling through interaction with, and modification of, secreted host derived factors.

## Results

### Effect of *E. coli* Nissle on 5-HT overflow

**Figure 1:**
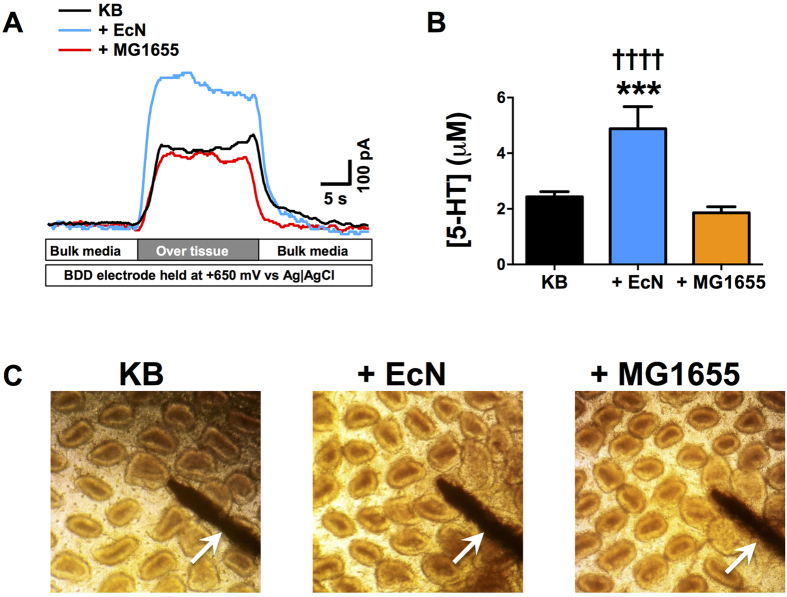
Measurement of extracellular 5-HT in ileal tissues treated with *E. coli* Nissle 1917 and MG1655. Ileal tissue samples and *E. coli* cultures were co-incubated for 1 h at 37 °C, 5% CO_2_, and extra cellular 5-HT levels measured in real-time using electrochemical methods (amperometry). (**A**) Example of electrochemical measurements obtained during experiments and converted to uM 5-HT based on standard curves constructed from known 5-HT concentrations. The BDD electrode was placed 0.1 mm over tissue. (**B**) Measurements of extracellular 5-HT from treated and untreated tissue sections. In all cases levels are normalized to surface area of the tissue. Data shown as mean ± S.E.M. (n = 4). (**C**) Example images of treated or untreated tissue sections used in experiments demonstrating intact epithelium following treatment and measurements. Arrows indicated the BDD electrode, and all images are ×10 magnification. **KB** - control samples incubated with Krebs buffer only; **+EcN**- samples treated with *E. coli* Nissle 1917; **+MG1655** - samples treated with *E. coli* MG1655. ***p ≤ 0.001 vs KB; ^††††^p ≤ 0.0001 vs MG1655.

In order to assess the effect of EcN on 5-HT overflow from EC cells we first treated *ex-vivo* ileal tissue with cultures of EcN, or the gastrointestinal commensal *E. coli* MG1655 as a control, and measured extra-cellular levels of 5-HT post treatment. In tissues treated with EcN we observed a significant elevation in extra-cellular levels of 5-HT, compared to untreated controls or MG1655 treated tissues (Fig. 1a,b). Microscopic examination of tissue treated with *E. coli* cultures confirmed that tissue remained intact and the observed elevation in 5-HT levels induced by EcN was not due to the disruption or degradation of the intestinal epithelium (Fig. 1c).

### The role of EcN K5 capsule in 5-HT overflow

**Figure 2:**
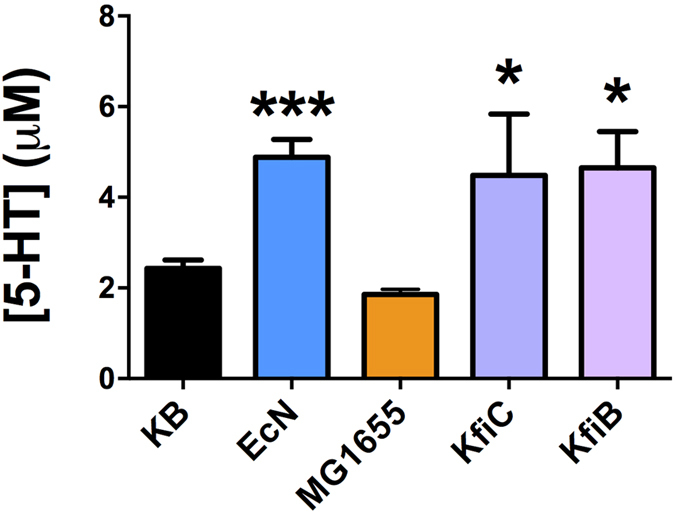
Role of *E. coli* Nissle 1917 K5 capsule in 5-HT overflow. The potential role of the EcN K5 capsule in 5-HT overflow was investigated using the isogenic K5 deficient mutants EcN∆*kfiB and* EcN∆*kfiC* (Nzakizwanayo *et al.*^46^). Ileal tissue samples were treated with live cell suspensions and extracellular 5-HT levels measured using amperometry as in Fig. 1. Data shown represent means ± S.E.M. (n = 4). **KB** - control samples incubated with Krebs buffer only; **EcN**- samples treated with *E. coli* Nissle 1917; **MG1655** - samples treated with *E. coli* MG1655; **KfiC** - samples treated with EcN∆*kfiC*; **KfiB**- samples treated with EcN∆*kfiB.* ***p ≤ 0.001 vs KB; *p ≤ 0.01 vs KB.

Given the distinct impacts of EcN and MG1655 on extra cellular 5-HT in our *ex-vivo* system, we next sought to understand how key differences in these gut-associated strains of *E. coli* may account for their differential effects on 5-HT overflow. Although structurally and biochemically similar, a major distinguishing feature of EcN and MG1655 is the composition of the outer polysaccharide capsule, with EcN generating a K5 type capsule compared to the MG1655 K12 type capsule. Since these outer surface structures are often the point of contact between host and bacterial cells, and in light of the established role of the EcN K5 in host-microbe interactions^45^,^46^, we hypothesized that the EcN K5 capsule may play a role in the observed EcN induced elevation of extra-cellular 5-HT. To test this, ileal tissue was treated with isogenic EcN capsule deficient mutants, EcN∆*kfiB* and EcN∆*kfiC,* in which core K5 biosynthesis genes are deleted^46^. These experiments showed K5 deficient mutants were still able to induce significantly elevated levels of extracellular 5-HT, comparable to those elicited by wild type EcN (Fig. 2), excluding a role of the K5 capsule in EcN mediated 5-HT overflow.

### Short chain fatty acid production is not involved in EcN mediated 5-HT overflow

**Figure 3:**
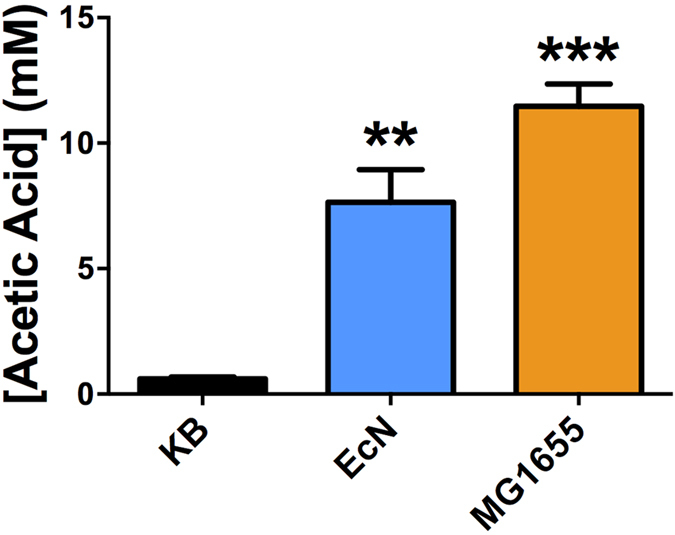
Role of short chain fatty acid production in EcN mediate 5-HT overflow. To determine if the capacity for production of short chain fatty acids (SCFA) was enhanced in EcN compared to MG1655, and relevant to EcN mediated 5-HT overflow, the levels of SCFA generated by each strain during growth in mucosal co-culture was measured. Bacterial cell suspensions in krebs buffer with ileal mucosal scrapings were incubated at 37 °C with 5% CO_2_ for 1 h, before measurement of SCFA by HPLC. Propionate and butyrate were not detected in this assay but both EcN and MG1655 generate acetate as show in charts. Data show means ± S.E.M. (n = 3). **P ≤ 0.01 ***P ≤ 0.001 Vs KB. **KB** - Krebs buffer only; **EcN** – *E. coli* Nissle 1917; **MG1655** – *E. coli* MG1655

Because the production of short chain fatty acids (SCFA) such as acetate, butyrate and propionate have been shown to stimulate 5-HT release *in vivo*^47^, and acetate is a key end product of *E. coli* growth in glucose containing media such as Krebs buffer, we next assessed the potential for the observed effects to be the result of enhanced SCFA production in EcN compared to MG1655. As expected, neither strain was able to generate detectable levels of butyrate or propionate, but both produced acetate under co-culture conditions with intestinal tissues (Fig. 3). However, under these conditions no significant differences in acetate production were observed between EcN and MG1655, indicating the elevated 5-HT release instigated by EcN was not attributable to acetate production.

### EcN modulates 5-HT overflow through interaction with secreted host-derived factors

**Figure 4:**
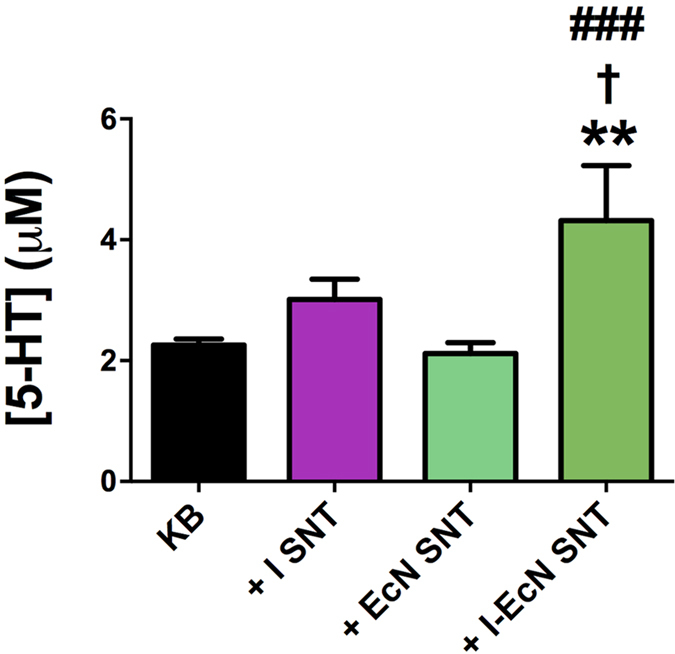
EcN modulates 5-HT bioavailabiliy through interaction with secreted host derived factors. The effect of secreted EcN or host derived factors on 5-HT overflow was determined by measuring 5-HT release after treatment of tissues with various culture or co-culture supernatants (at 37 °C with 5% CO_2_ for 1 h). Extracellular 5-HT was measured by amperometry as for Fig. 1. Data are means ± S.E.M. (n = 4), and measurements normalized to the surface area of tissue sections. **KB** - Krebs buffer only; **+I-SNT** – Tissue sections exposed to supernatant derived from untreated ileal tissue (incubated in Krebs buffer only); **+EcN-SNT –** Tissue exposed to supernatants derived from *E. coli* Nissle 1917 cultures grown in Krebs buffer; **+I-EcN- SNT** – Tissue exposed to supernatants from untreated ileal tissue incubated with EcN cell suspensions. **p ≤ 0.01 vs KB, ^†^p ≤ 0.05 vs I-SNT, ^###^p ≤ 0.001 vs EcN-SNT.

Recent studies have also indicated that a range of soluble metabolites generated by gut microbes increase 5-HT bioavailability in murine models, and include those derived from the modification of host generated compounds^43^. Therefore, to determine if the effect of EcN on extra-cellular 5-HT was attributable to factors that may be secreted by EcN; the result of direct interaction of EcN cells with ileal tissues; or due to the interaction of EcN with host derived factors, we next investigated the effect of a range of cell-free supernatants on 5-HT overflow in our *ex-vivo* co-culture system (Supplementary Fig. 1). These were derived from either: i) *E. coli* cultured in Krebs buffer alone (EcN-SNT); ii) Untreated ileal tissue incubated in Krebs buffer only (I-SNT); iii) Supernatants from untreated ileal tissue incubated with EcN cell suspensions prior to exposure of fresh tissue sections with derived cell-free supernatants (I-EcN-SNT). I-SNT recovered from untreated ileal tissues showed no significant impact on 5-HT levels when used to treat fresh tissue sections. Treatment of ileal tissue with EcN-SNT also resulted in no significant alteration in extra-cellular 5-HT, contrary to treatment with EcN cell suspensions (Fig. 4). However, when SNT recovered from ileal tissues were first incubated with EcN cells (I-EcN-SNT), before subsequent treatment of fresh tissue, 5-HT levels were significantly elevated (Fig. 4). These results point to an interaction of EcN with ileum-secreted factors to regulate 5-HT overflow in EC cells.

### Effect of *E. coli* Nissle on 5-HT synthesis and clearance

**Figure 5:**
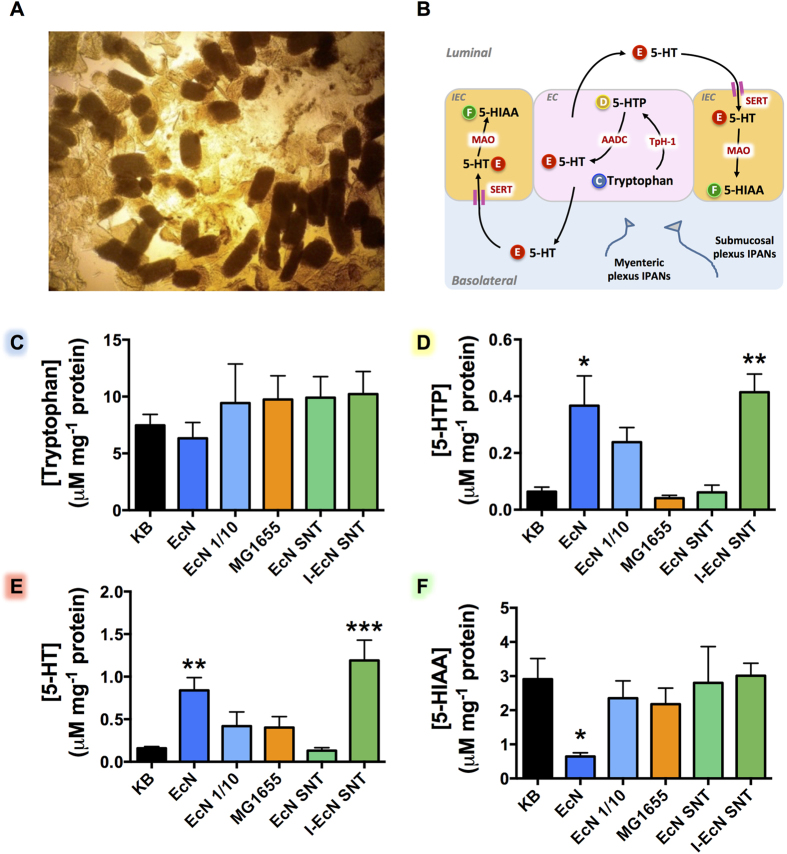
Neurotransmission process in ileal cells treated with *E. coli* Nissle 1917. To determine the impact of EcN treatment on the entire neurotransmission process, disaggregated intestinal villi (mucosal scrapings) were used to measure intracellular levels of 5-HT, precursors and metabolites following EcN treatment. Samples of Ileal mucosal scrapings were co-cultured with control Krebs buffer (KB), EcN cell suspensions (EcN), *E. coli* MG1655 cell suspensions (MG1655); EcN supernatants (EcN- SNT) or EcN modified ileal supernatants (I-EcN SNT) for 1 h at 37°C, 5% CO_2_, and post co-culture levels of neurochemicals were determined by HPLC. (**A**) Microscopic analysis of ileal mucosal cells after co-culture with live EcN, demonstrating intact villi structures post treatment. (**B**) Schematic of the serotonin pathway, highlighting major intermediates and key enzymes and transporters, in 5-HT synthesis and clearance. Compounds measured in these experiments are indicated by letters (**C,D,E,F**), and relate directly to data displayed in parts (**C,D,E,F**) of this figure. **Compounds:** 5HTP = 5-hydroxy-tryptophan; 5-HT = serotonin; 5-HIAA = 5-hydroxy-3-indoleacetic acid. **Enzymes & transporters:** TpH-1 = Tryptophan hydroxylase, catalyzes conversion of tryptophan to 5-HTP; AADC = Aromatic L-amino acid decarboxylase, converts 5-HTP to 5-HT in this pathway; SERT = Serotonin transporter, transports extra-cellular 5-HT into enterocytes; MAO = Monamine oxidase, catalyzes the metabolism of 5-HT to 5-HIAA in enterocytes. **Cell types:** IEC - Intestinal Epithelial Cell; EC - Enterochromaffin Cell; IPANs - Intrinsic Primary Afferent Neurones. (**C,D,E,F**) Levels of intracellular tryptophan; Intracellular 5-HTP; total intracellular and extracellular 5-HT; and intracellular 5-HIAA; respectively. Data are means ± S.E.M. (n = 4), and measurements normalized to the tissue mass by total protein. **KB** - Krebs buffer only; **EcN** – *E. coli* Nissle 1917; **EcN 1/10** – *E. coli* Nissle 1917 at 1/10 cell density of EcN; **MG1655** – *E. coli* MG1655; **EcN-SNT –** Supernatants derived from *E. coli* Nissle 1917 cultures grown in krebs buffer; **I-EcN- SNT** – Supernatants from untreated ileal tissue incubated with EcN cell suspensions. *p ≤ 0.05, **p ≤ 0.01, and ***p ≤ 0.001 vs KB.

Because the observed effect of EcN on extra-cellular 5-HT may be due to action at various stages of the serotonergic system (synthesis, release, clearance), we next investigated the impact of EcN on the 5-HT pathway as a whole. Following EcN treatment, both intracellular and extra-cellular levels of 5-HT, precursors, and metabolites were determined in dissociated ileal mucosa by HPLC (Fig. 5a,b). Intracellular levels of the 5-HT precursor tryptophan were not significantly different across the treated samples and controls (Fig. 5c). In contrast, intracellular levels of the tryptophan derived 5-HT intermediate, 5-Hydroxytryptophan (5-HTP), were shown to be significantly higher in tissues treated with both EcN cells and I-EcN-SNT (Fig. 5d). Commensurately, measurement of total 5-HT (intra and extracellular) indicated a >5-fold increase of 5-HT content in tissues treated with either EcN cells or with I-EcN-SNT, compared to untreated controls (Fig. 5e). Moreover, the stimulatory effect of EcN on 5-HTP and 5-HT was dose-dependent, and proportionally reduced when inoculum density of EcN was reduced 10-fold. The 5-HIAA content of dissociated mucosa was also significantly altered by EcN treatment, however, in this case distinct effects were observed when tissue was treated with EcN cells compared with I-EcN-SNT (Fig. 5f). In tissues treated with EcN cells 5-HIAA was significantly decreased, however, no significant alteration in 5-HIAA levels were observed when tissues were treated with I-EcN-SNT (Fig. 5f). No significant effect was elicited by the commensal MG1655 on the levels of any compound measured.

## Discussion

Recent studies have provided clear evidence that bacteria can modulate transmitter signalling in the GI tract, and in doing so influence local gut function, as well as aspects of systemic metabolism and potentially also mood and behavior^13^,^43^,^48^. Collectively, these studies highlight the potential for probiotic-based modulation of transmitter signalling systems to help tackle a wide range of disorders^13^,^43^,^48^,^49^,^50^. In the present study, we applied *ex vivo* co-culture models using murine ileum tissue, in conjunction with chemical and electroanalytical approaches, to determine the impact of EcN on the serotonergic system. The *ex vivo* co-culture system provides some important advantages in the initial evaluation of agents that modulate transmitter signaling in gut tissues. In particular, this permits the detailed and accurate measurements of the entire 5-HT transmission process directly in the mucosa, and in real-time, which is presently not possible *in vivo*. In contrast, the measurements of 5-HT possible during *in vivo* studies (from luminal or faecal contents) are unlikely to fully reflect the local mucosal situation, where the physiological effects of 5-HT are exerted and turnover takes place. The *ex-vivo* system allowed us to monitor both extra and intracellular levels of relevant metabolites directly in intact tissues, in response to defined “doses” of EcN, and although not without limitations (as covered in subsequent discussion), this system is generally considered to provide a good representation of the *in vivo* response.

This revealed that EcN is able to enhance bioavailability of 5-HT in a dose-dependent (bacterial density) manner. Notably the levels of EcN found to induce this elevated 5-HT overflow in our study are comparable to those found in commercial preparations, with Mutaflor capsules containing between 2.5 × 10^9^–10^10^ cfu of viable EcN (Ardeypharm GmbH). Furthermore, our data suggested that EcN may modulate 5-HT signalling at multiple points in the serotonin pathway, with evidence for distinct mechanisms working at different stages (synthesis and clearance).

In the case of the elevated 5-HT overflow elicited by EcN, this was not attributable to direct host-microbe interactions, or key components of the cell surface previously implicated in EcN probiotic effects (the K5 capsule). Instead, EcN mediated 5-HT overflow appears to be directed primarily by the interaction of EcN with exogenous factors secreted from host tissues. Compounds in cell-free supernatants of EcN have been previously found to mediate probiotic effects such as reinforcement of the intestinal mucosal barrier^51^,^52^,^53^, modulation of human colonic motility^54^, and regulation of immune functions^55^,^56^. Moreover, recent studies have identified a suite of soluble bacterial metabolites, generated by indigenous gut microbes, that increases 5-HT synthesis in EC cells^43^. These include metabolites that may be derived from interactions with host-generated substrates such as the SCFA butyrate, propionate, and acetate, as well as the secondary bile acid deoxycholate. However, our findings confirm that EcN like other *E. coli* strains does not generate appreciable levels of propionate or butyrate, and comparable levels of acetate to the non-serotogenic *E. coli* MG1655 under co-culture conditions, eliminating SCFA production by EcN as a source of the observed 5-HT elevation.

In terms of secondary bile acid production, this activity is also believed to be largely lacking in *E. coli*, and confined to members of the *Clostridiales*, which is congruent with the phylogenetic affiliation of indigenous 5-HT modulating organisms identified in other studies^43^,^57^,^58^. Our own confirmatory analyses suggests that both MG1655 and EcN encode genes with homology to those involved in secondary bile acid formation in other bacteria, albeit weak homology in most cases (Supplementary Table 1). Therefore, it is possible that EcN may be capable of generating secondary bile acids in our model system, but, it is unlikely that relevant levels of free primary bile acids (substrates for secondary BA formation) remain in the *ex-vivo* tissue sections after recovery and preparation. Furthermore, identifiable genes for bile acid de-conjugation (which is required before secondary bile acid formation can take place^57^,^59^), are absent from the EcN genome sequence (Supplementary Table 1), making secondary BA formation unlikely to explain our observations.

Other bacterial metabolites identified as potential regulators of 5-HT synthesis or overflow include tyramine, p-aminobenzoate (PABA), and α-tocopherol. Collectively the synthesis of these compounds are linked to the production of chorismate through the shikimate biosynthesis pathway^60^,^61^, although α-tocopherol is considered to be restricted to plants and photosynthetic bacteria, and therefore unlikely to be relevant in our experiments^60^. Of particular relevance to 5-HT signalling is the association of the shikimate pathway and chorismate biosynthesis with *de novo* generation of several aromatic amino acids, which include the tyramine precursor tyrosine, and also the 5-HT precursor tryptophan. Some bacterial species commonly found in the gut microbiome have also been shown to possess tryptophan decarboxylase activity and to generate the serotonergic potentiator tryptamine, which is known to induce 5-HT release from EC cells^62^. Tyramine is also a biogenic amine formed through the decarboxylation of the amino acid tyrosine, and can act as a neurotransmitter and stimulates chatecholamine release, influencing gut motility^43^,^63^.

Taken together, these observations afford the potential for a direct impact on 5-HT signalling through microbially derived tryptamine as well as tyramine. However, the lack of activity from EcN culture supernatants argues against the production of tryptamine and tyramine (at least at levels sufficient to influence 5-HT overflow), and available evidence supports a lack of tyrosine decarboxylase activity among *E. coli* species in general^62^. Our own survey of both EcN and MG1655 genome sequences, using recently described bacterial tryptophan decarboxylases of proven function^62^, did identify genes with weak homology to the *C. sporogenes* tryptophan decarboxylase in both strains (Supplementary Table 2). Despite this, the divergent impacts of MG1655 and EcN on 5-HT overflow do not support tryptamine production as responsible for the enhanced 5-HT bioavailability generated by EcN treatment.

Conversely, the production of folate is an essential biosynthetic pathway in *E. coli* and other bacteria, and bacteria in the gut are responsible for the generation of a significant fraction of the folate required by humans^61^,^64^. PABA is a key intermediate in this pathway, and although there is currently no clear physiological role for PABA in regulation of gut function or homeostasis, a possible impact on 5-HT bioavailability has been documented^43^,^64^. However, the lack of this pathway and production of the majority of precursors in mammals, along with the lack of 5-HT modulation exhibited by *E. coli* MG1655 in our experiments, would also cast doubt on changes in folate biosynthesis and generation of PABA as responsible for the observed impact of EcN on 5-HT bioavailability. This would require interaction with host-derived factors to influence folate biosynthesis in EcN but not MG1655, which remains a possible, if unlikely, explanation for the observed 5-HT modulation by EcN.

Taken together, our data clearly indicates that interaction between secreted host factors and EcN is required to generate the observed effects on 5-HT overflow, but provides little evidence to support the involvement of bacterial metabolites previously documented to influence 5-HT bioavailability (SCFA, secondary bile acids, tyramine, tryptamine, PABA, α-tocopherol)^43^, and the exact nature of this signal remains to be determined. Nevertheless, our results do indicate that the basic mechanism through which 5-HT levels are elevated is similar to that described for bacteria in recent studies^43^. Formation of the 5-HT precursor 5-HTP is catalysed by Tph-1, which converts tryptophan to 5-HTP, and is the rate-limiting step in 5-HT synthesis and turnover in gut tissues^65^,^66^,^67^. Our analysis of EcN impact on the overall serotonin circuit, through measurement of the major precursors and metabolites, showed that EcN was able to increase the levels of intracellular 5-HTP in a dose dependant manner (in contrast to the commensal *E. coli* MG1655). This points to an increase in Tph-1 activity in EcN treated tissues, ultimately leading to an increase in 5-HT synthesis, which has also been reported as the mechanism through which members of the indigenous gut microbiome increase 5-HT bioavailability^43^.

It is also notable that no significant differences in background tryptophan levels were observed in these experiments. Because tryptophan is the substrate for Tph-1, an increase in the activity of this enzyme and the wider 5-HT synthesis machinery, would be expected to lead to a commensurate reduction in background levels of tryptophan. This is supported by observations of elevated background tryptophan levels in serum and faeces of germ free animals where 5-HT synthesis is reduced^43^, and such changes in tryptophan concentration could be expected to be more pronounced in the closed *ex-vivo* system we have utilised in this study.

In contrast, our data appear more in keeping with conflicting reports of the relationship between tryptophan concentration and 5-HT synthesis, which describe elevated levels of tryptophan in plasma of germ free animals when 5-HT levels are also elevated in various tissues^13^,^44^. The ability of *E. coli* to synthesise tryptophan *de novo* may in part explain the stability of levels observed in our *ex-vivo* models, by offsetting increased utilisation when 5-HT synthesis is enhanced. However, since elevated 5-HTP was not observed for MG1655 treated tissues, despite comparable levels of tryptophan being detected in these experiments, it is unlikely that this explanation fully accounts for our observations, if at all, and further work will be required to understand the exact factors leading to the stability of tryptophan levels in our experiments, and also if this translates to the *in vivo* setting.

Our data also point to effects of EcN on 5-HT clearance. Typically, extra cellular 5-HT released into the lumen is cleared by uptake into enterocytes *via* the serotonin transporter SERT, and converted into the major metabolite 5-HIAA by the action of monoamine oxidase^25^,^26^. This continual clearance is required for completion of the serotonin circuit and termination of the biological effects of released 5-HT. In treatments that elevate extracellular 5-HT, we would therefore predict commensurate increases in levels of 5-HIAA. However, in tissues treated with EcN cells levels of 5-HIAA were significantly reduced (indicating reduced 5-HT clearance), while I-EcN-SNT treated tissues showed no significant difference in 5-HIAA compared to untreated controls.

The lack of significant changes to 5-HIAA in I-EcN-SNT treated tissues, despite elevated 5-HT levels, may have several explanations. These include the potential for the total 5-HT measured in these experiments to primarily reflect intracellular 5-HT, a saturation of SERT activity effectively limiting the rate of clearance, or an inhibitory effect on 5-HT uptake by SERT (again restricting the maximal level of clearance observed). The latter scenario is also congruent with the significant reduction in 5-HIAA observed in tissue sections treated with EcN cells (since supernatants will also contain cellular debris including cell envelope components), but suggests that cell-cell interaction is required for EcN to fully exert these effects, and raises the potential for a direct interaction and interference with SERT. Conversely, it is possible that EcN is able to modulate the activity of monoamine oxidase (MAO) to reduce the formation of 5-HIAA. Nonetheless, the intracellular location of MAO coupled with the significant reduction of 5-HIAA by EcN cells specifically, would suggest interaction between EcN and surface associated proteins such as SERT to be a more likely scenario.

In summary, our data highlight the ability of EcN to increase the bioavailability of 5-HT in intestinal tissues, *via* modulation of several distinct aspects of the serotonergic system (synthesis and clearance). These findings may have important implications for the role of probiotics such as EcN in the management of intestinal disorders, and the broader range of extra-cellular maladies in which 5-HT signaling plays a role. Notably, the potential increase in 5-HT afforded by EcN and potential impact on SERT are of interest in light of the long history of using selective serotonin reuptake inhibitors to treat mood disorders. This is particularly pertinent with the emerging role of the gut microbiome in homeostasis of the gut-brain-microbiome axis, and the potential for probiotic manipulation of this community to play an important role in the future treatment of mood disorders^13^,^48^.

In addition, studies of patients with constipation predominant IBS (IBS-c) have pointed to reductions in EC cell number and 5-HT production in gut tissues. This is hypothesized to perturb communication with the enteric nervous system, reducing intestinal motility and leading to constipation^36^,^68^. As such EcN mediated elevation of mucosal 5-HT could have therapeutic benefit in management of IBS-c, or other functional disorders where 5-HT reduction is implicated. On the contrary, conditions where elevated 5-HT has been linked to disease pathology, such as some types of IBD associated with loss or inactivity of SERT^69^, there may be potential for EcN administration to exacerbate the underlying disease state, highlighting the need for a clear understanding of host-probiotic interactions to ensure the most appropriate and effective use of probiotics. Notable in this context are the lack of effects we observed in experiments with the well characterised gut commensal *E. coli* MG1655^70^ pointing to modulation of 5-HT as a specific “probiotic” trait of EcN rather than a property of gut-associated *E. coli* in general.

Finally, although a powerful approach, the *ex-vivo* co-culture system used here is not without limitations, which should be kept in mind when interpreting these data, and particularly when considering any possible impact on host health. Perhaps of most importance is the likely perturbation of the intestinal microbiota and luminal milieu in the *ex-vivo* system, both of which may contribute to regulation of gut transmitter systems and influence the response to treatments with agents such as EcN. The wider impact of EcN 5-HT modulation on host health will clearly require the conduct of carefully controlled *in vivo* studies, including those evaluating long-term impact of 5-HT modulation, and the data presented here should now provide a sound platform to facilitate the design of these subsequent investigations.

## Materials and Methods

### Bacterial strains and cultures

*E. coli* Nissle 1917 (EcN; Ardeypharm GmbH, Herdecke, Germany), its K5 capsule mutants, EcN∆*kfiB and* EcN∆*kfiC*^46^, and *E. Coli* K12 strain MG1655 (CGSC7740)^70^ were used in this study. All strains were routinely grown in Lauria-Bertani (LB) broth (Fisher Scientific, UK) overnight at 37 °C with shaking (150 rpm). Cells were harvested by centrifugation, re-suspended in sterile Krebs buffer solution pH 7.4 (mM: 117 NaCl, 4.7 KCl, 2.5 CaCl_2_, 1.2 MgCl_2_, 1.2 NaH_2_PO_4_, 25 NaHCO_3_, and 11 glucose) and used in co-cultures in a final concentration of 2 × 10^9^ Cfu/mL (EcN) or 2 × 10^8^ Cfu/mL (EcN 1/10).

### Collection of intestinal tissue

All tissue samples used in this study were obtained from three-month-old male C57BL/6 mice. All procedures were carried out according to U.K. Home Office regulations and were approved by the University of Brighton Ethics Committee. Mice were euthanized *via* CO_2_ then cervical dislocation, the abdomen was subsequently opened and intestines removed. A section of the distal ileum (2 cm from caecum) was dissected and placed in ice-cold oxygenated (95%, O_2_, 5% CO_2_) Krebs buffer solution pH 7.4.

### Ileal tissue preparation for measurement of serotonin and neurotransmission processes

Two protocols were utilised in order to investigate the influence of EcN on extracellular 5-HT and intracellular precursors and metabolites, as previously described by Lwin & Patel^71^ and Parmar *et al.*^72^. For both protocols, ileal sections were initially divided into 1 cm long segments, transferred to an inverted pre-chilled glass Petri dish, opened longitudinally along the mesenteric border, and laid out with the mucosal surface facing up giving ~1 cm^2^ segments. The flattened tissue was subsequently rinsed twice with Krebs buffer solution to remove any visible faecal matter.

For measurement of extracellular 5-HT using amperometry, rinsed tissue segments were transferred to a 6-well plate, each well containing 2 mL ice-cold oxygenated Krebs solution for further co-culture experiments. For intracellular monitoring of 5-HT precursors and metabolites, rinsed tissue segments were gently scrapped to remove the mucosal layer using a scalpel. The resulting tissue scrapings were transferred to a chilled 1.5 mL- Eppendorf tube and gently aggregated using a plastic microfuge pestle to get individual villi disaggregated from the supporting connective tissue. This suspension was then vortex for 1 min and fractions were transferred to a 6-well plate for further co-culture experiments.

### Preparation of cell free supernatants

An overview of cell free supernatants utilised in this study is provided in Supplementary Fig. 1. ***EcN Supernatants (EcN-SNT):*** Supernatants derived solely from *E. coli* Nissle 1917 cultures (EcN-SNT) were obtained by recovery of EcN cultures from LB broth cultures by centrifugation, washing of recovered cells with PBS, and re-suspension at 2 × 10^9^ Cfu/mL in Krebs buffer. These cell suspensions were incubated at 37 °C, 5% CO_2_ for 1 h. Supernatants were then collected by centrifugation (13,000 *g*, 5 min) and filtration using a Whatman 0.45 μm pore-size filter, before use directly in experiments. ***Ileum Supernatants (I-SNT):*** Supernatants obtained from untreated ileum sections were recovered following incubation of tissue in Krebs buffer (37 °C, 5% CO_2_ for 1 h), followed by centrifugation and filtration as above. ***Ileum EcN modified Supernatants (I-EcN-SNT):***To obtain supernatants exposed to both Ileum and EcN cells independently, EcN cells recovered from LB broth cultures were suspended in I-SNT preparations described above to a final density of 2 × 10^9^ Cfu/mL, and incubated for a further hour (37 °C, 5% CO_2_). The resulting ileum-EcN-SNT was recovered by centrifugation and filtration as above, before use in further experiments.

### Amperometric measurement of extracellular 5-HT in co-culture experiments

Oxygenated ileal tissues were co-incubated with *E. coli* cell suspensions (in Krebs buffer at 2 × 10^9^ Cfu/mL) or cell free supernatants (see above) in 6-well plates. Co-cultures were incubated for 1 h at 37 °C, 5% CO_2,_ and controls included ileal sections with Krebs buffer only. After co-incubation, tissue samples were pinned in a Sylgard® (Dow Corning, UK) lined Teflon recording chamber and perfused with warm (37 °C) Krebs solution at a flow rate of 2 mL/min. Tissues were perfused for 10 min prior to commencing a series of measurements. For continuous amperometric recordings of 5-HT overflow, measurements were carried out using a BioStat™ multi-channel potentiostat (ESA Biosciences, Inc, USA). A three-electrode system was utilised with a stainless steel auxiliary electrode, a Ag|AgCl (3 M KCl) reference electrode, and a 76 μm boron-doped diamond (BDD) electrode which served as the working electrode. The BDD microelectrode potential was held over the tissue at +650 mV vs Ag|AgCl which was sufficient to oxidize serotonin at a mass transfer limited rate^73^,^74^. Using a micromanipulator, the BDD microelectrode was positioned several centimetres away from the mucosa for several seconds for background recordings. For recordings from tissues sections, the electrode was positioned 0.1 mm over the tissue for 20 s, where reproducible oxidation currents were recorded. This procedure was repeated 5 times for each tissue.

### HPLC measurement of 5-HT and intermediates in co-culture experiments

Mucosal scrapings were inoculated with EcN cell suspensions (at 2 × 10^9^ Cfu/mL in 250 μL final volume Krebs buffer) or supernatants (see above) in 12-well plates then incubated for 1 h at 37 °C, 5% CO_2._ Controls included mucosal scrapings with Krebs buffer only, as well as EcN in Krebs buffer without addition of mucosal scrapings. After co-incubation, samples were immediately transferred to prechilled 1.5 mL sterile Eppendorf tubes and homogenised in 250 μL of ice-cold 0.1 M HClO_4_ by vortexing. The samples were then centrifuged at 13,000 g for 10 min at 4 °C and the resultant supernatant was analysed for 5-HT, its precursors and metabolites, using HPLC with Electrochemical Detection ED as described by Parmar *et al.*^72^. The HPLC apparatus consisted of a Jasco HPLC pump (Model: PU-980) and Rheodyne manual injector equipped with a 20 μl loop. A Kinetic^®^ ODS 2.6 μm 150 mm × 4.6 mm i.d. analytical column with a guard column (Phenomenex^®^, Macclesfield, UK) was employed. The HPLC system was run at a flow rate of 1.0 mL min^−1^. CHI630B potentiostat (CH Instruments, Austin, TX, USA) was used to control the detector voltage and record the current. A 3 mm glassy carbon electrode (flow cell, BAS) served as the working electrode and was used with a Ag|AgCl reference electrode and a stainless steel block as the auxiliary electrode. Amperometric recordings were carried out, where the working electrode was set at a potential of +850 mV vs. Ag|AgCl reference electrode. Control and data collection/processing were handled through the CHI1001A software. The stock buffer for the mobile phase comprised of the following: 0.1 M sodium acetate, 0.1 M citric acid and 27 μM disodium ethylene-diamine-tetra-acetate (EDTA). This was then buffered to pH 3.0. The mobile phase was prepared with the stock buffer mixed with methanol in the ratio of 8:2 (v/v) and degassed after mixing. Standard solutions of 5-HTP, 5-HT, tryptophan and 5-HIAA were prepared in 0.1 M perchloric acid (Sigma Aldrich). Each of the standard solutions were prepared on the day of analysis and stored at 4 °C prior to injection. A calibration plot was obtained for each of the chemicals at a concentration range of 0.01–10 μM. The peak areas obtained from chromatographic analysis of all injected samples were converted to concentrations utilising the calibration responses. The concentration of each chemical was normalized by the total protein content using the Bradford method to account for differences in mucosal biomass between samples. Control samples of bacteria in Krebs buffer only without mucosal scrapings were used to determine average bacterial protein content, which was subtracted from all co-cultured samples.

### Measurement of short chain fatty acids from EcN and MG1655

Production of short chain fatty acids (SCFA) was measured after growth of *E. coli* strains with intestinal mucosal scraping under co-culture conditions (as described above), and filtration (0.2-μm-pore-size filter), and used for analysis of derivatives SCFA by reversed phase HPLC (RP-HPLC) developed by Schiffels *et al.*^75^. Analyses were performed using a Waters 2695 Alliance Separation Module, equipped with a quaternary gradient pump, column oven autosampler (Kinetex), and a Waters 2487 Dual Absorbance UV/Vis Detector and a PE Nelson 900 series Interface. The data was collected *via* TotalChrom navigator software (PerkinElmer, Shelton, CT). Mobile phase A consisted of 20 mM triethylamine, 20 mM ammonium acetate, adjusted to pH 4.7 with formic acid. Mobile phase B was acetonitrile. Total SCFAs were extracted by addition of 0.2 g of NaCl and 100 μL of concentrated HCl to 300 μL aliquots, followed by 800 μL of diethyl ether containing 35 mM hexanoic acid internal standard. Following centrifugation (1 min at 12,000 *g*), 200 μL of the obtained ether phase was recovered for analysis, and treated with 200 μL of oxalyl chloride solution [250 mM oxalyl chloride in N,N-dimethyl formamide/acetonitrile 1:100 (v/v)]. Tubes were incubated at room temperature for 5 min then 800 μL of derivatization reagent (50 mM 4-nitrophenol in 500 mM pyridine/acetonitrile) was added to produce 4-nitrophenyl esters of SFCAs. After derivatization, 20 μL of each SCFA standard and sample solution were injected onto a C_18_ column (Fortis 150 mm ×3 mm, 3 μM; Fortis Technologies Ltd, Cheshire, UK) at 40 °C, with detection at λ = 295 nm. The following binary gradient (A%/B%) was applied: 20/80 (hold 1 min) to 0/100 (in 6 min) back to 20/80 (in 5 min, hold 5 min). Flow rate was 0.6 mL/min throughout.

### Data analysis

Peak areas from chromatograms of neurochemicals were normalised to the protein content of the sample. The normalised data were expressed as micromolar concentration of the neurochemical being tested per milligram protein of the mucosal-bacterial sample (μM mg-1 protein). The difference in the baseline to peak current observed when the BDD microelectrode was placed over the tissue during amperometry recordings were monitored and the resultant current was converted to concentration using a pre-calibrated response to 5-HT. ANOVA was used for comparison between grouped samples, assuming populations were Gaussian (*H*_*0*_: means are equal, 95% confidence interval level). Whenever *H*_*0*_ was rejected (P < 0.05), the Bonferroni post-test (post-hoc) to correct for multiple comparisons was employed. Data were analysed using GraphPad Prism V5.03.

## Additional Information

**How to cite this article**: Nzakizwanayo, J. *et al.* *Escherichia coli* Nissle 1917 enhances bioavailability of serotonin in gut tissues through modulation of synthesis and clearance. *Sci. Rep.* **5**, 17324; doi: 10.1038/srep17324 (2015).

## Supplementary Material

Supplementary Information
