# Supplementary material for: Escherichia coli Nissle 1917 enhances bioavailability of serotonin in gut tissues through modulation of synthesis and clearance
Source: Sci Rep. 2015 Nov 30;5:17324. doi: 10.1038/srep17324 (PMC4663480; doi:10.1038/srep17324)
Supplement: Supplementary Information [file srep17324-s1.pdf]

Supplementary information for:

Escherichia coli Nissle 1917 enhances bioavailability of serotonin in gut tissues through modulation of synthesis and clearance.

Jonathan Nzakizwanayo[1], Cinzia Dedi[1], Guy Standen[1], Wendy M Macfarlane[1], Bhavik A Patel[1,*], and Brian V Jones[1,2,*]

[1] School of Pharmacy and Biomolecular Sciences, University of Brighton, Brighton, BN2 4GJ, United Kingdom.

[2] Queen Victoria Hospital NHS Foundation Trust, East Grinstead, RH19 3DZ, United Kingdom.

* **Correspondence** to B.A.P (email: B.A.Patel@brighton.ac.uk) or B.V.J. (email: B.V.Jones@brighton.ac.uk)

Supplementary Figure 1

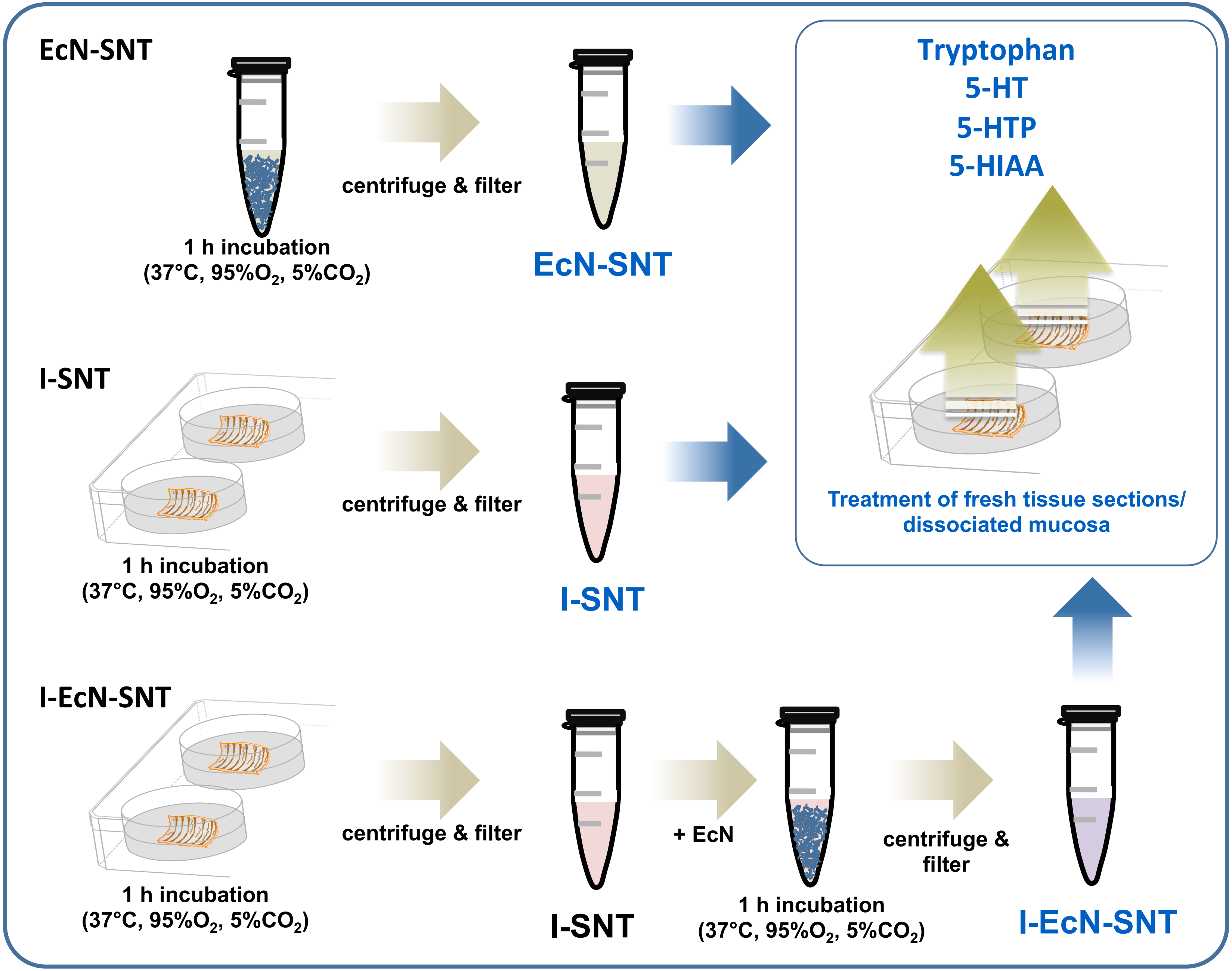

Figure Supplementary 1: Overview of cell free supernatants used in this study. To determine the if effects of EcN on 5-HT overflow were the attributable to EcN secreted products; interaction of EcN cells with host epithelium; or the interaction between EcN and host derived secreted factors, a range of cell free supernatants were generated and their impact on 5-HT overflow measured. **EcN-SNT:** Cell free supernatants derived from incubation of EcN cells only in Krebs buffer under co-culture conditions. **I-SNT**: Cell free supernatants derived from incubation of ileal tissue sections only in Krebs buffer under co-culture conditions. **I-EcN-SNT**: Cell free supernatants derived from pre-incubation of I-SNT with EcN cells, prior to recovery of cell free supernatants.

Supplementary Table 1: Survey of *E. coli* MG1655 and Nissle 1917 genomes for genes associated with bile acid transformation.

Strain	Homologues of genes involved in bacterial bile acid modification[1]				
	Query[a]	e-value	Identity	query coverage	Position in Reference Genome[b]
Nissle 1917	*Eschericia coli* HB101 7 Alpha Hydroxysteroid Dehydrogenase (1943534)	1.05e^{-127}	98.80%	100.00%	101393-100629 CAPM01000056
	Clostridium hylemonae DSM15053 BaiF (ACF20987)	2.09e^{-67}	38.40%	92.92%	72230-73417 CAPM01000108
	Clostridium scindens 7 alpha hydroxysteroid dehydrogenase (AAB611510)	6.87e^{-24}	34.50%	98.12%	127342-126569 CAPM01000141
	Clostridium sordellii 7 alpha hydroxysteroid dehydrogenase (AAA53556)	1.54e^{-28}	34.00%	95.88%	46341-45598 CAPM01000047
	Clostridium scindens 3 alpha hydroxysteroid dehydrogenase (AAC45414)	6.26e^{-24}	33.70%	72.69%	444119-443550 CAPM01000141
	Clostridium hylemonae DSM15053 BaiH (ACF20989)	8.20e^{-68}	32.40%	78.27%	178284-179834 CAPM01000069
	Clostridium scindens BaiL (ACF20980)	7.39e^{-11}	32.00%	52.26%	51681-52058 CAPM01000056
	Clostridium hylemonae DSM15053 BiaA (ACF20977)	3.01e^{-21}	30.80%	72.69%	4624-4082 CAPM01000033
	Clostridium scindens BaiCD (AAC45411)	4.01e^{-29}	28.90%	51.02%	130020-131045 CAPM01000056
	Clostridium hylemonae DSM15053 BaiB (ACF20984)	3.02e^{-14}	28.20%	39.69%	182815-183444 CAPM01000056
	Clostridium scindens bile acid coenzyme A ligase (AAC45410)	8.16e^{-29}	28.00%	66.15%	283619-282585 CAPM01000056
	Clostridium hylemonae DSM15053 BaiK (ACF20982)	3.34e^{-24}	26.60%	76.30%	391426-390410 CAPM01000141
	Clostridium hylemonae DSM15053 BaiCD (ACF20985)	3.40e^{-29}	26.50%	51.72%	53602-52568 CAPM01000092
	Clostridium scindens BaiK (ACF20979)	5.33e^{-25}	26.00%	86.96%	395521-394313 CAPM01000141
	Helicobacter pylori B8 7 alpha hydroxysteroid dehydrogenase (YP 003729151)	5.01e^{-16}	25.90%	96.18%	183639-182899 CAPM01000136
MG1655	*Eschericia coli* HB101 7 Alpha Hydroxysteroid Dehydrogenase (1943534)	2.32e^{-128}	99.60%	100.00%	1698040-1697276
	Clostridium hylemonae DSM15053 BaiF (ACF20987)	1.22e^{-67}	37.90%	95.52%	39100-37880
	Clostridium sordellii 7 alpha hydroxysteroid dehydrogenase (AAA53556)	1.44e^{-28}	34.00%	95.88%	1150676-1151419
	Clostridium hylemonae DSM15053 BaiH (ACF20989)	5.30e^{-67}	32.20%	78.27%	3231647-3233197
	Clostridium scindens BaiL (ACF20980)	8.03e^{-20}	31.00%	86.42%	2983084-2982506
	Clostridium scindens bile acid coenzyme A ligase (AAC45410)	7.62e^{-29}	28.00%	66.15%	1889116-1888082

[1] An extensive collection of sequences, predicted or confirmed to be involved in bile acid metabolism (Jones *et al.* 2008)[57], were used as queries to search *E. coli* MG1655 (Riley *et al.* 2006) and Nissle 1917 (Cress *et al.* 2013) genome sequences using tBlastn (Altshul *et al.* 1990). Only hits displaying a maximum e-value of 1e^{-05} and a minimum of 25% identity to query sequences were considered valid and retained. Results were further processed to identify the top hit by bit score matching queries with valid hits. Where distinct queries aligned with the same region of the reference genome, these were resolved to a single hit based on best bit score. No valid hits to query sequences involved in bile acid de-conjugation (bile salt hydrolases or choloylglycine hydrolases) were obtained from these searches with valid hits limited to query sequences derived from enzymes involved in formation of secondary bile acids.

[a] Query sequences producing best hits to reference sequence genomes. Accession numbers are provided in parentheses.

[b] The position of retained hits in reference sequence genomes is reported as nt coordinates. For Nissle 1917 hits the relevant contig number is also provided.

Supplementary Table2: Survey of *E. coli* MG1655 and Nissle 1917 genomes for genes associated with tryptophan decarboxylase activity.

Strain	Homologues of genes involved in tryptophan decarboxylase activity[1]				
	Query	e-value	Identity	query coverage	Position in Reference Genome
**Nissle 1917**	*Clostridium sporogenes* CLOSPO_02083 (EDU35915.1)	7.54e^{-07}	27.9%	27.34%	148-480 CAPM01000046
**MG1655**	*Clostridium sporogenes* CLOSPO_02083 (EDU35915.1)	5.96e^{-07}	31.4%	19.66%	366668-3666761

[1] Sequences from bacterial enzymes derived from indigenous gut microbes recently confirmed to exhibit tryptophan decarboxylase activity (Williams *et al.* 2014)[62], were used as queries to search *E. coli* MG1655 (Riley *et al.* 2006) and Nissle 1917 (Cress *et al.* 2013) genome sequences using tBlastn. Only hits displaying a maximum e-value of 1e^{-05} and a minimum of 25% identity to query sequences were considered valid and retained.

[a] Query sequences producing best hits to reference sequence genomes. Accession numbers are provided in parentheses.

[b] The position of valid hits in reference sequence genomes is reported as nt coordinates. For Nissle 1917 hits the relevant contig number is also provided.

Supplementary References

Altschul, S.F., Gish W., Miller, W., Myers, E. W., Lipman, D. J. Basic local alignment search tool. *J. Mol. Biol.* **215,** 403–410 (1990).

Cress, B. F., Linhardt, R. J. & Koffas, M. A.Draft Genome Sequence of *Escherichia coli* Strain Nissle 1917 (Serovar O6:K5:H1). *Genome Announc* **1,** e0004713 (2013). doi: 10.1128/genomeA.00047-13.

Riley, M., *et al. Escherichia coli* K-12: a cooperatively developed annotation snapshot-2005. *Nucleic Acids Res.* **34**, 1-9 (2006)
